# Ultrasound shear wave elastography imaging of common carotid arteries in patients with Spontaneous Coronary Artery Dissection (SCAD)

**DOI:** 10.1007/s40477-021-00627-2

**Published:** 2022-01-15

**Authors:** Fahad F. Al-mutairi, Abtehal Al-hussaini, Anne-Marie Marsh, Nilesh Samani, Gerry McCann, David Adlam, Emma M. L. Chung, Kumar V. Ramnarine

**Affiliations:** 1 Department of Diagnostic Radiology, Faculty of Applied Medical Sciences, King Abdulaziz University, Jeddah, Saudi Arabia; 2 Department of Cardiovascular Sciences, University of Leicester, Leicester, UK; 3 National Institute for Health Research (NIHR) Leicester Biomedical Research Centre, Glenfield Hospital, Leicester, UK; 4 Department of Medical Physics, University Hospitals of Leicester NHS Trust, Leicester, UK; 5 Medical Physics Department, Guy’s and St Thomas’ NHS Foundation Trust, London, UK

**Keywords:** Carotid artery, Young’s modulus, Stiffness, Ultrasound shear wave elastography, Spontaneous coronary artery dissection

## Abstract

**Background:**

Shear wave elastography (SWE) is emerging as a valuable clinical tool for a variety of conditions. The aim of this pilot study was to assess the potential of SWE imaging of the common carotid arteries (CCA) in patients with spontaneous coronary artery dissection (SCAD), a rare but potentially life-threatening condition, hypothesized to be linked to changes in vessel wall elasticity.

**Methods:**

Ultrasound shear wave elastography (SWE) estimates of artery wall elasticity were obtained from the left and right CCAs of 89 confirmed SCAD patients and 38 non-dissection controls. SWE images obtained over multiple cardiac cycles were analysed by a blinded observer to estimate elasticity in the form of a Young’s Modulus (YM) value, across regions of interest (ROI) located within the anterior and posterior CCA walls.

**Results:**

YM estimates ranged from 17 to 133 kPa in SCAD patients compared to 34 to 87 kPa in non-dissection controls. The mean YM of 55 [standard deviation (SD): 21] kPa in SCAD patients was not significantly different to the mean of 57 [SD: 12] kPa in controls, *p* = 0.32. The difference between groups was 2 kPa [95% Confidence Interval − 11, 4].

**Conclusions:**

SWE imaging of CCAs in SCAD patients is feasible although the clinical benefit is limited by relatively high variability of YM values which may have contributed to our finding of no significant difference between SCAD patients and non-dissection controls.

## Introduction

Arterial dissection of the major arteries is caused by the development of a false lumen within the tunica media of the artery. Clinical sequelae depend on the site of dissection, but can include myocardial infarction (for SCAD) [1], and stroke, in the event of dissection of the carotid or vertebral (cervical) arteries.

The underlying causes of arterial dissection are not well understood, but have been hypothesized to be linked to changes in vessel wall biomechanics. Dissection events often affect young to middle-aged people with few risk factors for atherosclerotic cardiovascular disease [2, 3]. In SCAD, pregnancy, in particular, appears to place otherwise healthy women at increased risk of dissection, especially in the late third trimester and early post-partum period [4]. Identification of non-invasive imaging biomarkers for identification of patients at risk of dissection would be highly beneficial.

Although the pathogenesis of dissection is currently unclear, SCAD has been demonstrated to be associated with remote arteriopathies, such as fibromuscular dysplasia (FMD) in the renal and carotid arteries [5, 6]. Previous research suggests that increased arterial stiffness might make arteries more vulnerable to dissection [7]. Calvet et al. previously examined arterial wall motion over the cardiac cycle to estimate Young’s Modulus (YM) in a small sample of 32 patients with spontaneous cervical artery dissection and found abnormal elastic properties of the carotid artery in patients with dissection [8].

This study adopts the newer technique of ultrasound shear wave elastography (SWE) to directly quantify CCA stiffness in SCAD patients compared to non-dissection controls. SWE imparts focused ultrasound ‘push pulses’ within tissue, generating a propagating acoustic ‘shear wave’. The velocities of the resultant shear waves are measured and analysed by ultrafast ultrasound imaging to display a real-time 2D (elastogram) map of tissue stiffness [9]. The feasibility of quantifying YM in vessels has been established through in vitro and ex vivo studies [10, 11]. Moreover, recent clinical studies investigating vascular applications of SWE in easily accessible carotid arteries have shown promising results, highlighting the potential clinical value of SWE in vessel wall and plaque characterisation [12–14].

This study assessed carotid arterial wall stiffness in patients with confirmed SCAD for blinded comparison with non-dissection controls. The aim of the study was to assess whether YM estimation using SWE imaging of the common carotid artery (CCA) might be useful as a diagnostic biomarker relating to the risk of arterial dissection, which would also provide a better understanding of SCAD pathophysiology.

## Methods

### Participants and study protocol

This study was sponsored by the University Hospitals of Leicester NHS Trust under NHS Research Ethics Committee Health Research Authority approval (REC reference 14/EM/0056). All participants provided written, fully informed, consent.

Included patients had angiographically confirmed SCAD diagnosed by at least two experienced clinicians. 89 SCAD patients (median age 48 years) and 38 non-dissection controls (median age 43 years) were enrolled in this study. As part of the study protocol, body mass index (BMI), and blood pressure (BP) were measured. Both systolic and diastolic pressure in both groups were investigated. Demographic information (age and sex) and risk factors for cardiovascular disease (hypertension, hypercholesterolemia, diabetes mellitus, and family history of cardiovascular disease) were also recorded.

Ultrasound scans of the left and right common carotid arteries (CCA) were performed in SCAD patients and non-dissection controls using a SWE ultrasound scanner equipped with a 15–4 MHz linear array probe (Axiplorer, Supersonic Imagine, France). The following SWE scanner settings were selected to optimise image quality: maximum acoustic power output, mid-range smoothing (6), persistence off, penetration mode, and colour map range up to 300 kPa. Approximately 10 s of cine-loop visualising longitudinal sections of the artery located 2 cm proximal to the carotid bulb were acquired.

**Fig. 1:**
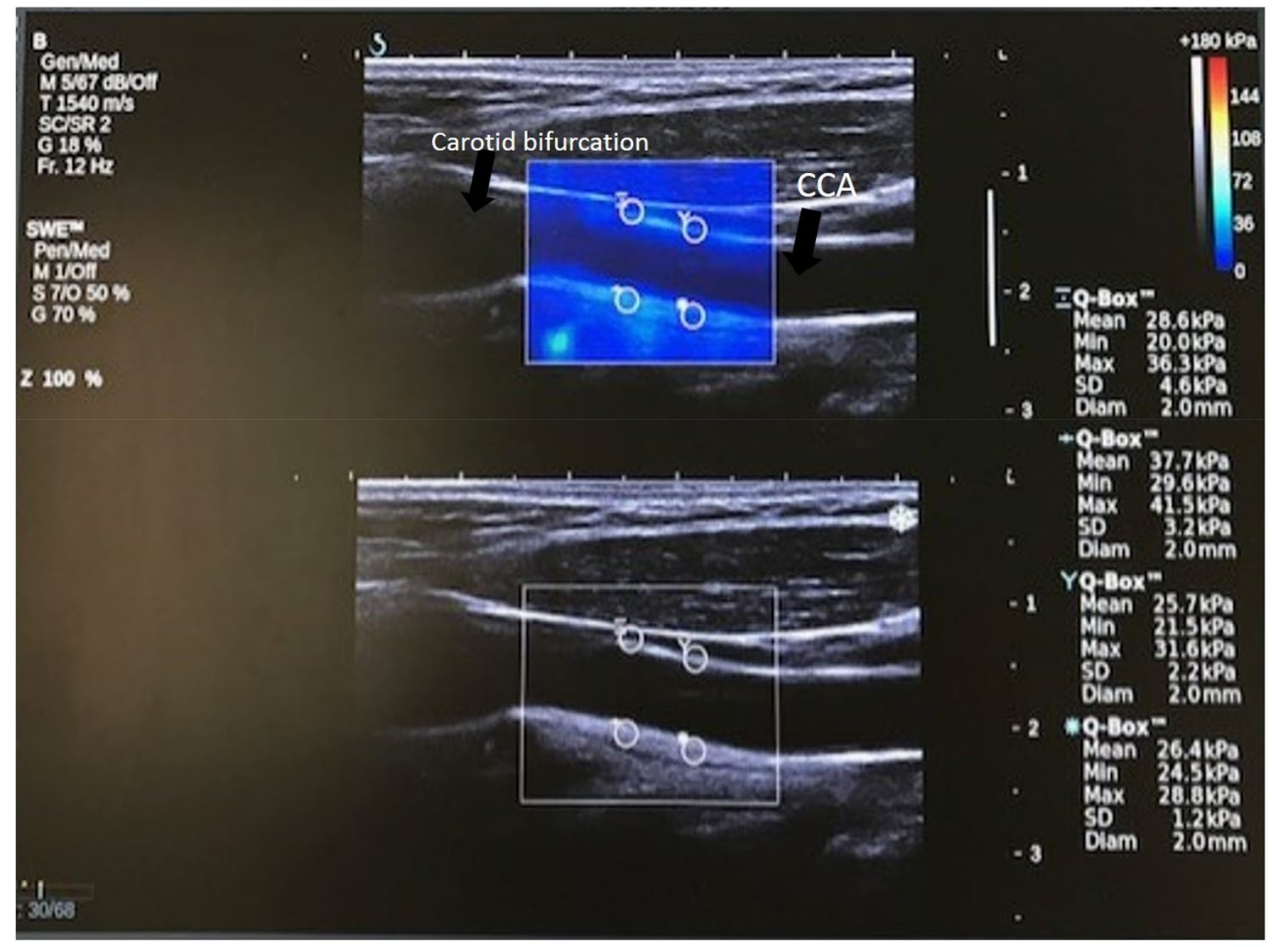
Example of a single CCA image frame showing placement of 4 ROIs for estimation of mean YM. Top image shows SWE image box and bottom B-mode image

SWE scans were analysed retrospectively by an experienced observer, who was blinded to whether data came from a SCAD subject or non-dissection control. Arterial wall stiffness was quantified using the Aixplorer’s built-in analysis software to measure mean Young’s modulus from several 2 mm Regions of Interest (ROIs). From the acquired cine-loops, the first two SWE frames were discarded to enable the SWE acquisition to settle. Five consecutive frames were then analysed to estimate mean YM within each ROI. Two ROIs were placed on the anterior wall and two on the posterior wall of the left and right carotid arteries, as shown in Fig. 1. This resulted in a total of 40 YM measurements per participant (4 ROIs * 5 frames * 2 sides). Criteria for inclusion of measurements in further statistical analyses were: (1) good image quality; (2) complete filling of the shear wave elastogram; (3) adequate cine-loops (over a minimum of five frames). Exclusion criteria included: (1) poor image quality; (2) insufficient shear wave elastogram filling; (3) cine-loops with fewer than five SWE frames. Recordings were assessed for quality by an independent assessor who was blinded to whether images originated from patients or controls.

### Statistical analysis

Data were analysed using GRAPHPAD PRISM^®^ version 7 software (Prism, California, USA). Underlying statistical assumptions regarding normality were tested using a Shapiro–Wilks test. Demographic factors for SCAD patients and non-dissection controls were compared using Student’s *t*-test for normally distributed data, and non-parametric tests (Wilcoxon and Mann–Whitney tests) for non-normally distributed variables. As YM estimates were normally distributed, an independent samples Student’s *t*-test was used to compare average elasticity estimates for SCAD compared to those of non-dissection controls, and to estimate 95% confidence limits on the mean difference between groups. A *p* value of *p* < 0.05 was considered statistically significant.

## Results

### Demographic and clinical characteristics

A total of 127 carotid examinations from 38 non-dissection controls (35 females: 3 males) and 89 SCAD patients (87 females: 2 males) were performed. The demographic and clinical characteristics of SCAD and non-dissection control groups are summarised and compared in Table 1. Significant differences (*p* < 0.0001) were observed for age and blood pressure parameters. On average, SCAD patients were 5 years older than the control group (SCAD: median age 48 years [range: 43 to 53 years]; controls: median age 43 years [range 36 to 49 years], *p* < 0.001). Systolic and diastolic pressures were both significantly lower in the SCAD group, with a mean (SD) systolic pressure in SCAD patients of 119 (16) mmHg compared to 130 (18) mmHg in non-dissection controls (*p* = 0.001); the mean (SD) diastolic pressure of 72 (12) mmHg in SCAD patients was also significantly lower than the 78 (11) mmHg in non-dissection controls (*p* < 0.009), see Table 1.

**Table 1:** Comparison of demographic characteristics and YM estimates for SCAD patients versus non-dissection controls

	SCAD (*n* = 89)	Controls (*n* = 38)	Difference
Sex, female: male	87:2	35:3	*p* = 0.32^1^
Age, years (range)	48 (43–53)	43 (36–49)	*p* < 0.001^3^
BMI, kg/m^2^ (range)	25 (22–29)	25 (21–29)	*p* = 0.99^3^
Diastole, mmHg (SD)	72 (12)	78 (11)	− 6.4 [95% CI − 11, − 1.7], *p* = 0.009^2^
Systole, mmHg (SD)	119 (16)	130 (18)	11 [95% CI − 18, − 4.6], *p* = 0.001^2^
Hypertension, *n* (%)	18 (20%)	N/A	–
Hypercholesterolemia, *n* (%)	7 (8%)	N/A	–
Diabetes Mellitus, *n* (%)	7 (8%)	N/A	–
Family History, *n* (%)	56 (63%)	N/A	–
Young’s Modulus, kPa (SD)	55 (21)	57 (12)	2 [95% CI: − 11, 4], *p* = 0.32^2^

^1^Chi-squared test, ^2^*t*-test, ^3^Mann–Whitney test

*SD* standard deviation

### CCA mean YM in SCAD patients compared to non-dissection controls

**Fig. 2:**
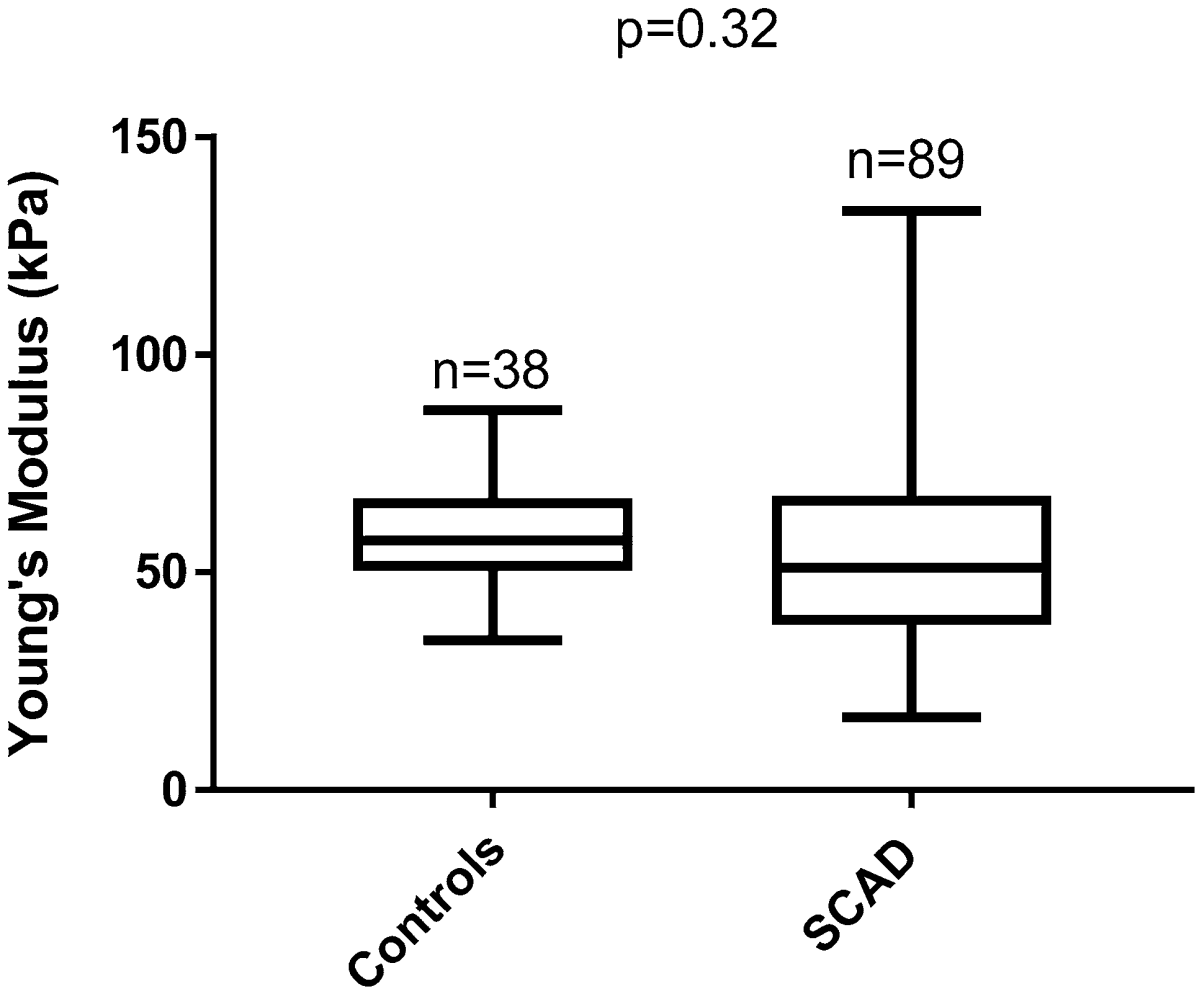
Box and Whisker plot of mean YM estimates in the CCAs of SCAD patients and non-dissection controls

Measurements from all RoIs; left and right carotid arteries, anterior and posterior walls, across 5 frames for each subject, were averaged to provide a single estimate of the mean YM for each individual participant. Mean YM estimates were highly variable, ranging from 17 to 133 kPa in SCAD patients and between 34 and 87 kPa in controls. CCA YM estimates were confirmed to be normally distributed, with a mean (SD) YM of 55 (21) kPa in SCAD patients compared to 57 (12) kPa in non-dissection controls (Fig. 2). An unpaired (independent samples) Student’s *t*-test confirmed no significant difference in mean YM between SCAD and control subjects (*p* = 0.32). The difference between groups was estimated to be 2 kPa [95%CI − 11, 4] suggesting that any difference between SCAD patients and non-dissection controls, if one exists, must be less than 11 kPa.

## Discussion

In this study, we perform a first SWE comparison of YM estimates of common carotid artery stiffness in SCAD patients compared to non-dissection controls, with the aim of providing a better understanding of the pathophysiology of SCAD. The mean YM of the CCA in patients with confirmed SCAD was estimated to be within 2 [95% CI − 11 to 4] kPa of non-dissection controls, with no significant difference between groups, *p* = 0.34. The controls were slightly younger and with higher blood pressure than the SCAD group. This is a limitation of our study as a similar control group, at least for age, should be matched closer to the SCAD group. Nevertheless, our main finding of no difference in stiffness and high variability of YM estimates mean this is unlikely to have influenced these key findings. This result is at variance with a previous smaller study by Calvet et al*.* that identified a significantly higher CCA stiffness in patients with spontaneous cervical artery dissection [8]. However Calvet et al. estimated stiffness through analysis of vessel wall compliance using ultrasound echo-tracking, a different technique to SWE so direct comparison is difficult. In our study, we directly measured YM using SWE and found a wide range of YM estimates in subjects and no significant difference in mean YM between SCAD patients and controls. This may suggest that major artery stiffening is not a significant factor in the pathogenesis of arterial dissection in SCAD patients. However this requires further work as the hypothesis that YM measured in the common carotid artery is indicative of that of the coronary artery is not proven.

Calvet et al*.* hypothesized that conducting arteries of patients with spontaneous cervical artery dissection undergo a higher level of circumferential wall stress than those of control subjects and found a 14% increase in CCA circumferential wall stress which was linked to higher risk for dissection. They found that carotid arteries, but not aorta and radial artery, displayed abnormal elastic properties in patients with spontaneous cervical artery dissection and concluded that higher stiffness of carotid wall material and circumferential wall stress could increase the risk of dissection in these patients. Interestingly, abnormal elastic properties were observed at the site of the CCA in patients with spontaneous cervical artery dissection independently of the site of dissection (ipsilateral or contralateral, carotid or vertebral artery) and the number of dissected arteries.

Our study was limited as only local carotid stiffness was assessed and only patients with coronary dissection were included in the study while other sites of dissection were not considered. We found that SWE measurements generate a wide range of YM estimates making practical utility of this approach in clinical practice limited. Adjusting for the power of our study, the 95% confidence limits of − 11 to + 4 kPa for the difference between groups is narrow compared to measurement variability, and includes zero signaling there may be no difference. SWE was initially developed for assessing bulk homogeneous tissues, such as the liver, therefore, several challenges and limitations are associated with measurement of YM in arteries. The relationship between shear wave velocity and YM is based on theoretical assumptions of constant density, homogeneity, isotropy, and incompressibility, which may not be valid in vessels. In particular, slender vessels support Lamb wave propagation requiring a different theoretical model for YM estimation [9, 14, 15]. Our study is limited by the high variability observed in SWE CCA YM measurements. Further work would be beneficial for understanding sources of YM variability to determine whether these reflect true heterogeneity in tissue stiffness, or are a result of limitations of SWE when applied to vessels.

## Conclusions

SWE imaging of CCAs in SCAD patients is feasible although the clinical benefit is limited by relatively high variability of YM values which may have contributed to our finding of no significant difference between SCAD patients and non-dissection controls.

## Data Availability

The data that support the findings of this study are available from the corresponding author, [FA], upon reasonable request.
